# A change in the bacterial community of spider mites decreases fecundity on multiple host plants

**DOI:** 10.1002/mbo3.743

**Published:** 2018-10-11

**Authors:** Yu‐Xi Zhu, Yue‐Ling Song, Ary A. Hoffmann, Peng‐Yu Jin, Shi‐Mei Huo, Xiao‐Yue Hong

**Affiliations:** ^1^ Department of Entomology Nanjing Agricultural University Nanjing China; ^2^ School of BioSciences Bio21 Institute, The University of Melbourne Melbourne Victoria Australia

**Keywords:** 16S rRNA, fecundity, host plant, spider mite, symbiotic bacterial

## Abstract

Bacterial symbionts may influence the fitness of their herbivore hosts, but such effects have been poorly studied across most invertebrate groups. The spider mite, *Tetranychus truncatus,* is a polyphagous agricultural pest harboring various bacterial symbionts whose function is largely unknown. Here, by using a high‐throughput 16S rRNA amplicon sequencing approach, we characterized the bacterial diversity and community composition of spider mites fed on five host plants after communities were modified following tetracycline exposure. We demonstrated that spider mite bacterial diversity and community composition were significantly affected by host plants and antibiotics. In particular, the abundance of the maternally inherited endosymbionts *Wolbachia* and *Spiroplasma* significantly differed among spider mites that were reared on different plant species and were completely removed by antibiotics. There was an overall tendency for daily fecundity to be lower in the mites with reduced bacterial diversity following the antibiotic treatment. Our data suggest that host plants and antibiotics can shape spider mite bacterial communities and that bacterial symbionts improve mite performance.

## 1 INTRODUCTION

Three‐way interactions between plants, arthropods, and microbes are ubiquitous and complex (Biere, Tack, & Bennett, 2013; Shikano, Rosa, Tan, & Felton, 2017). Arthropod‐associated bacteria can profoundly influence the outcome of plant–arthropod interactions (Frago, Dicke, & Godfray, 2012; Giron et al., 2017). For example, arthropod‐associated microorganisms can aid in the exploitation of plant resources by essential nutrient supplementation (Chandler, Wilkinson, & Douglas, 2008), degradation of complex structural metabolites (Berasategui et al., 2017; Hammer & Bowers, 2015; Hansen & Moran, 2014), and defense manipulation (Chung et al., 2013; Su et al., 2015).

Conversely, the symbiotic bacterial communities of arthropod herbivores are affected by host genotype (Brady et al., 2014), host plant (Wilkinson, Adams, Minto, & Douglas, 2001), and antibiotic treatments (Breeuwer, 1997; Staudacher et al., 2017). Host plants influence the diversity and abundance of symbionts of aphids (Zhang, Cao, Zhong, Godfray, & Liu, 2016), whiteflies (Pan et al., 2014; Su et al., 2016), and other arthropod herbivores (Morrow, Frommer, Shearman, & Riegler, 2015; Strano, Malacrino, Campolo, & Palmeri, 2017). For example, in polyphagous or oligophagous aphids, including *Aphis gossypii* (Jones, Bressan, Greenwell, & Fierer, 2011), *Aphis citrcidus* (Guidolin & Cônsoli, 2016), *Aphis fabae* (Chandler et al., 2008), and *Acyrthosiphon pisum* (Tsuchida, Koga, Shibao, Matsumoto, & Fukatsu, 2002), both primary and secondary symbionts are affected by the plant type on which the aphids feed. Host plants shape the diversity and abundance of larval gut symbiotic bacteria of the Colorado potato beetle, *Leptinotarsa decemlineata*, and thus affect the insect's ability to manipulate plant defenses in *Solanum* hosts (Chung et al., 2017). Similarly, antibiotic treatment can also alter the bacterial communities of herbivores (Lehman, Lundgren, & Petzke, 2009; Zouache, Voronin, Tran‐Van, & Mavingui, 2009); in particular, antibiotic treatment significantly influences the relative abundance of *Wolbachia*,* Spiroplasma,* and/or *Cardinium* in the spider mite *Tetranychus urticae* (Staudacher et al., 2017). Antibiotics are routinely used to eliminate some endosymbionts from a wide range of insect species (Li, Floate, Fields, & Pang, 2014; Wilkinson, 1998). Although previous studies showed that host plants and antibiotic may be important factors in shaping the bacterial community of several herbivorous arthropod species, little is known about the effect of host plants and antibiotic treatment on the entire bacterial communities of spider mites.

There are over 1,000 species of spider mites (*Tetranychus* sp.), including several that are economically important pests damaging agricultural crops and ornamental plants, with approximately 0.9 billion Euro being spent annually for their control worldwide (Migeon, Nouguier, & Dorkeld, 2010; Van Leeuwen, Tirry, Yamamoto, Nauen, & Dermauw, 2015). Spider mites host a large community of symbiotic bacteria, including facultative endosymbionts such as *Wolbachia*,* Rickettsia*,* Cardinium,* and *Spiroplasma* (Chaisiri, McGARRY, Morand, & Makepeace, 2015; Zélé, Santos, Olivieri, et al., 2018; Zhang, Chen, Yang, Qiao, & Hong, 2016), which manipulate host reproduction via various phenotypic effects (Engelstädter & Hurst, 2009; Moran, McCutcheon, & Nakabachi, 2008; Werren, Baldo, & Clark, 2008). Host plants that lower *Wolbachia* prevalence in natural *T. urticae* populations may also lower egg hatchability (Zélé, Santos, Godinho, & Magalhães, 2018), pointing to the potential for three‐way interactions between microbes, plants, and spider mites.

Among spider mites, *Tetranychus truncatus* is a highly polyphagous species found on over 60 host plant species, including economically important crops such as bean, cotton, cucumber, tomato, and eggplant (Bolland, Gutierrez, & Flechtmann, 1998). *Tetranychus truncatus* is the dominant mite species in China and has diverse host plants (Zhang et al., 2013). We previously demonstrated that *T. truncatus* harbor various endosymbiotic bacteria, including *Wolbachia*,* Cardinium,* and *Spiroplasma* (Zhang, Chen, et al., 2016), and affect host reproduction through cytoplasmic incompatibility (CI) (Zhang, Yang, Zhu, & Hong, 2018; Zhao, Zhang, & Hong, 2013). Infection prevalence of *Wolbachia* in *T. truncatus* natural populations is related to ecological factors, such as host plant, temperature, and climate (Zhu et al., 2018), but the interaction between these factors and host fitness is not clear.

In this study, we explore host plant and antibiotic influences on spider mite symbiotic bacterial communities and performance under controlled environmental conditions. Recent developments in sequencing technologies and molecular tools have enhanced opportunities to characterize the microbial diversity associated with spider mites (Sugio, Dubreuil, Giron, & Simon, 2015). We used a high‐throughput 16S rRNA amplicon sequencing procedure to investigate whether antibiotic treatment and host plant influence the composition and structure of *T. truncatus* bacterial communities and host performance. The results highlight roles of host plant and antibiotics in shaping the bacterial community of herbivores and highlight impacts of bacterial diversity on mite fecundity.

## 2 MATERIALS AND METHODS

### 2.1 Plants

Five host plant species were used in this study: *Gossypium hirsutum* L. cultivar Nannong 10 (cotton), *Cucumis sativus* L. cultivar Lufeng (cucumber), *Solanum lycopersicum* L. cultivar Hezuo 903 (tomato), *S. melongena* L. cultivar Suquqi (eggplant), and *Phaseolus vulgaris* L. cultivar Sucaidou 11 (bean). Seeds of the five plants were purchased from Jiangsu Academy of Agricultural Sciences. Plants were germinated in soil for 2 weeks. Individual plants were grown in plastic pots in a climate‐controlled room at 25 ± 1°C, 60% relative humidity, and under a 16‐hr light: 8‐hr dark photoperiod. Plants were used for experiments at the 4‐ to 6‐leaf stage.

### 2.2 Spider mite antibiotic treatment and rearing

**Figure 1:**
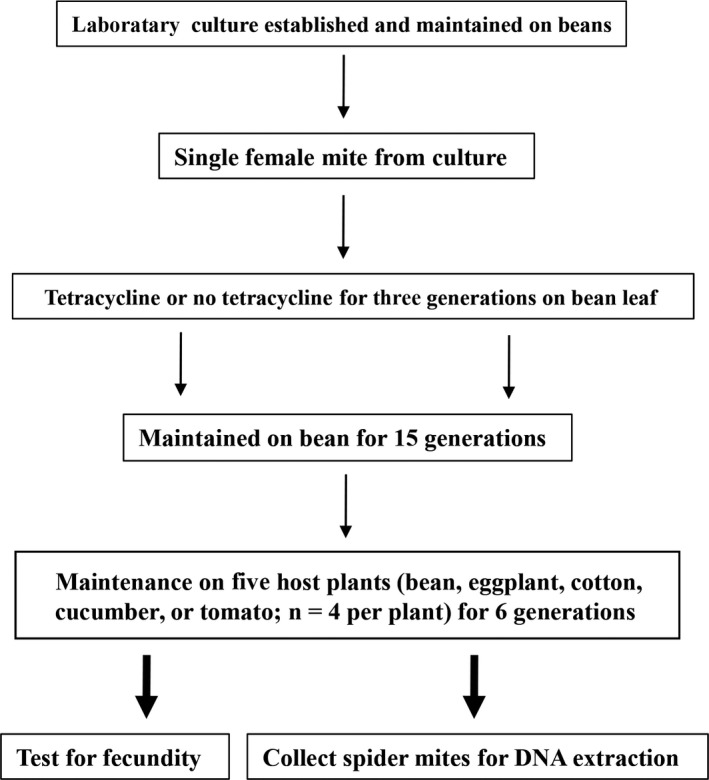
Overview of experimental procedure describing the different rearing condition used to compare the performance of antibiotic‐untreated and antibiotic‐treated spider mites after maintenance on different host plant and collection of samples for DNA extraction

Spider mites were originally collected from bean (*Phaseolus vulgaris* L.) leaves in Hohhot, Inner Mongolia, northeast China in August 2014. Mites were reared on detached bean leaflets in a climate‐controlled room at 25 ± 1°C, 60% relative humidity, and a light:dark (L:D) photoperiod of 16:8 hr. Individuals used to establish various mite strains were all derived from one adult female to minimize genetic variation between lines (Figure 1).

To investigate the effect of antibiotics on microbial communities associated with *T. truncatus* spider mites, lines of mites were treated with antibiotics. For the antibiotic treatment, 30 adult females mites were reared on bean leaf disks on cotton wool soaked with tetracycline solution (0.1%, w/v) for three generations. Untreated control mites were reared on leaf disks placed on water‐saturated cotton wool. After treatment, lines were maintained in a mass‐rearing environment without antibiotics for approximately 15 generations to establish *T. truncatus* lines on different host plants. The treated lines are referred to as AB‐T and the untreated lines as AB‐UT.

### 2.3 Establishment of *T. truncatus* lines on different host plants

To characterize the microbial communities associated with spider mites that feed on different hosts, lines were established by transferring adult female mites (ca. 400) of both the AB‐UT and AB‐T lines from bean to cotton, tomato, cucumber, or eggplant. Four independent lines on each plant have been created. Lines were maintained on detached leaves from these hosts for six generations in a climatically controlled environment at 25 ± 1°C with 60% relative humidity and a 16L:8D photoperiod. More than 200 mites were transferred at each generation.

To generate mites for experiments, we used 4‐ to 6‐day‐old adult females from each strain to produce eggs on the adaxial surface of detached plant leaflets placed on water‐soaked cotton. After 6 hr of egg production, all mites were removed from the leaflets. The eggs were allowed to hatch and mature in a climate‐controlled room for another 13 days; this was done to obtain spider mites of the same age. The adult female mites were then collected for the performance assay and for DNA extraction (Figure 1).

### 2.4 Spider mite performance assay

To investigate the effect of host plants on spider mite performance, we assessed spider mite fecundity on different plants using the method described by Staudacher et al. (2017) with minor modifications. The mite lines were maintained on detached leaves from five different plants in a climate room (25 ± 1°C, 16 hr:8 hr, light:dark, 60% RH). For the performance assay, leaf disks of bean, cucumber, cotton, and eggplant (diameter ca. 3 cm) and tomato leaflet (at least 4 cm in length) were placed on a cotton bed soaked in water. Five adult female mites (2 ± 0.25 d) were placed on each leaf disk (or leaflet), 24 leaf disks (or leaflets) per line (treated or not with antibiotics) and per plant species (*n* = 5 species). After 4 days, the number of eggs produced by mites was recorded using a stereomicroscope.

### 2.5 DNA extraction, quantitative real‐time polymerase chain reaction (qPCR), and 16S rRNA amplicon sequencing

Fifteen adult female spider mites from each of the replicated plants belonging to the five different plant species were pooled to form one sample for DNA extraction. DNA was extracted from each sample using the E.Z.N.A.^®^ Soil DNA Kit (Omega Bio‐Tek, Norcross, GA, USA) according to manufacturer's protocols.

The *Wolbachia* and *Spiroplasma* densities in spider mite samples were estimated by qPCR as described previously (Zhang et al., 2018). Briefly, we used primers designed to amplify a 141‐bp fragment of *wsp* from *Wolbachia* (wQF1, 5′‐GAGCAGCGAATGTAAGCAATC‐3′, and wQR1, 5′‐AATAACGAGCACCAGCATAAAG‐3′) and a 141‐bp fragment of *16S rRNA* from *Spiroplasma* (sQF1, 5′‐TGTAGTTCTCAGGGA TTGTTTTCTC‐3′, and sQR1, 5′‐CGCTTCCACCATCGCTCTT‐3′). The PCR products of primers specific for *wsp* from *Wolbachia* and 16S rRNA from *Spiroplasma* were amplified by conventional PCR; then, the PCR products were purified using the AxyPrep TM DNA Gel Extraction Kit (Axygen) and cloned into a pEASY‐T1 vector (TransGen Biotech, Beijing, China). A standard curve was generated using a serial dilution of plasmids containing one copy of the target sequence. Absolute quantification of *wsp* and *16S rRNA* copy number was calculated using threshold values (*C*_t_).

The V3–V4 region of the 16S rRNA gene was amplified from each sample using the primer pair 341F (5′‐CCTAYGGGRBGCASCAG‐3′) and 806R (5′‐GGACTACNNGGGTATCTAAT‐3′). PCR was performed in a 25 μl volume that contained 12.5 μl 2× Taq Master Mix (Vazyme Biotech, China), 0.5 μl primer (20 μM each), and 1 μl of DNA, or ultrapure water for the PCR‐negative controls. The PCR conditions were as follows: 95°C for 5 min, followed by 27 cycles of 95°C for 30 s, 55°C for 30 s, and 72°C for 45 min, and a 72°C final extension for 10 min. PCR product quality was verified by gel electrophoresis. Amplicons were extracted from 2% agarose gels and purified using the AxyPrep DNA Gel Extraction Kit (Axygen Biosciences, Union City, CA, USA) according to manufacturer's instructions and quantified using QuantiFluor™‐ST (Promega, USA). Purified PCR products were quantified by Qubit^®^3.0 (Life Invitrogen), and 24 amplicons with different barcodes were mixed equally. DNA concentration was adjusted to 25–35 ng/μl per sample before sequencing. All DNA samples were sent for sequencing, except for those of the treated line 4 on bean and line 3 on cucumber and the untreated line 3 on cotton, because the DNA concentration of three samples was less than required by the criteria. The pooled DNA product was used to construct an Illumina paired‐end library following Illumina's genomic DNA library preparation procedure. Then, the amplicon library was paired‐end (2 × 250 bp) sequenced on an Illumina HiSeq 2500 platform (Shanghai Biozeron Co., Ltd) using standard protocols.

### 2.6 Sequence processing and analyses

Sequences were provided as adapter‐clipped fastq files and analyzed in Quantitative Insights into Microbial Ecology (QIIME), which is a standard pipeline for microbial community analysis (Caporaso et al., 2010). Raw fastq files were demultiplexed and quality‐filtered using QIIME 1.17 with the following criteria: (a) The 250‐bp reads were truncated at any site with an average quality score <20 over a 10‐bp sliding window, and truncated reads that were shorter than 50 bp were discarded; (b) exact barcode matches, two‐nucleotide mismatches in primer matching, and reads that contained ambiguous characters were removed; and (c) only sequences that overlap longer than 10 bp were assembled based on their overlapping sequences. Reads that could not be assembled were discarded.

Operational taxonomic units (OTUs) were clustered with 97% similarity cutoff using UPARSE 7.1 (https://drive5.com/uparse/), and chimeric sequences were identified and removed using UCHIME. The phylogenetic affiliation of each 16S rRNA gene sequence was analyzed with RDP Classifier (https://rdp.cme.msu.edu/) against the Silva 16S rRNA database using a confidence threshold of 70% (Amato et al., 2013). To avoid bias, OTUs (<0.1% abundance) were excluded from subsequent analysis. Rarefaction analysis was generated using Mothur 1.21.1 to determine Good's coverage, Chao 1, and Simpson and Shannon diversity indices (Schloss et al., 2009).

### 2.7 Statistical analyses of bacterial community

All statistical analyses were carried out in R ver 3.3.1 (R Development Core & Team, 2016).

Diversity of the bacterial communities in the samples was determined by computing Simpson and Shannon indices, while species richness was estimated through counting OTUs or computing ACE (abundance‐based coverage estimator) and Chao 1 indices (Hill, Walsh, Harris, & Moffett, 2003; Hughes, Hellmann, Ricketts, & Bohannan, 2001; Shannon, 1948; Simpson, 1949). To determine whether diversity measures were significantly different between samples from the different host/antibiotic treatments, we used two‐way ANOVAs after validation of the normal distribution of the residuals.

To determine whether feeding in different host plants or/and antibiotics caused major changes in community structure, a Bray–Curtis dissimilarity matrix was calculated and analysis of molecular variance (AMOVA) was used. Multi‐response permutation procedures (MRPP) analyses were also used to compare community composition between samples from the different treatments. Variation in bacterial taxonomic composition among samples was visualized using principal coordinates analyses (PCoA). PCoA was performed using the R package “vegan.”

To test the effect of antibiotics and/or host plant species on mite oviposition, we constructed a general linear model with two factors, antibiotics and host plant, treated as a fixed factor, and average number of eggs per females per day as response variables, which were firstly verified to follow normal distributions. If interaction terms were insignificant, mite oviposition was subjected to a one‐way ANOVA. The R package “lsmeans” was used for multiple comparisons. Sequence counts of 10 OTUs (*Wolbachia*,* Spiroplasma*,* Halomonas*,* Acinetobacter*,* Pelagibacterium*,* Pseudomonas*,* Comamonas*,* Paucibacter*,* Cloacibacterium*, and *Sphingobium*) and *wsp* and *16S rRNA* copy numbers follow a quasipoisson‐distributed, one‐way ANOVA were performed to detect the different of relative abundance of those OTUs in AB‐UT and AB‐T spider mite among five host plant species, respectively.

## 3 RESULTS

### 3.1 Overview of *T. truncatus* bacterial communities

Analyses of 16S rRNA amplicon sequences yielded a total of 865,578 reads after quality check, with an average of 23,394 sequences per sample. The majority of the rarefaction curves approached saturation, which indicated that our sampling depth accurately characterized the bacterial diversity of the majority of these samples (Supporting Information Figure S1).

**Figure 2:**
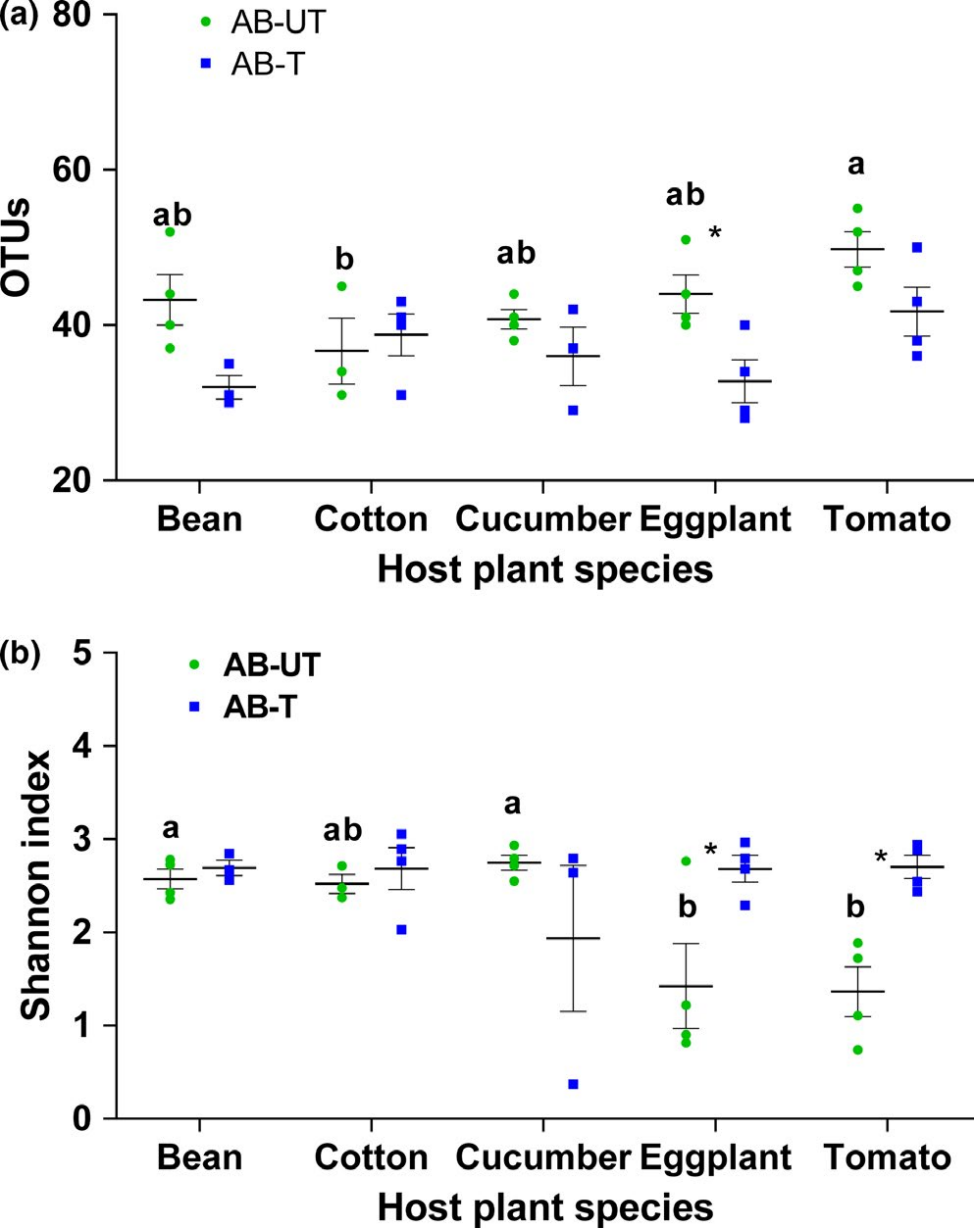
Alpha diversity indexes without singletons from antibiotic‐untreated and antibiotic‐treated spider mites that were reared for six generations on different host plants. (a) OTUs; (b) Shannon index; (c) Simpson index; and (d) Chao 1 index. Horizontal lines indicate the mean (±SE) of biological replicates. Superscripts (a, b) above horizontal lines indicate significant differences between antibiotic‐untreated mites that were reared on different host plants (*p* < 0.05). “*” represents significant difference between antibiotic‐untreated and antibiotic‐treated spider mites on the same host plant (*p* < 0.05). n.s.: not significant

**Figure 3:**
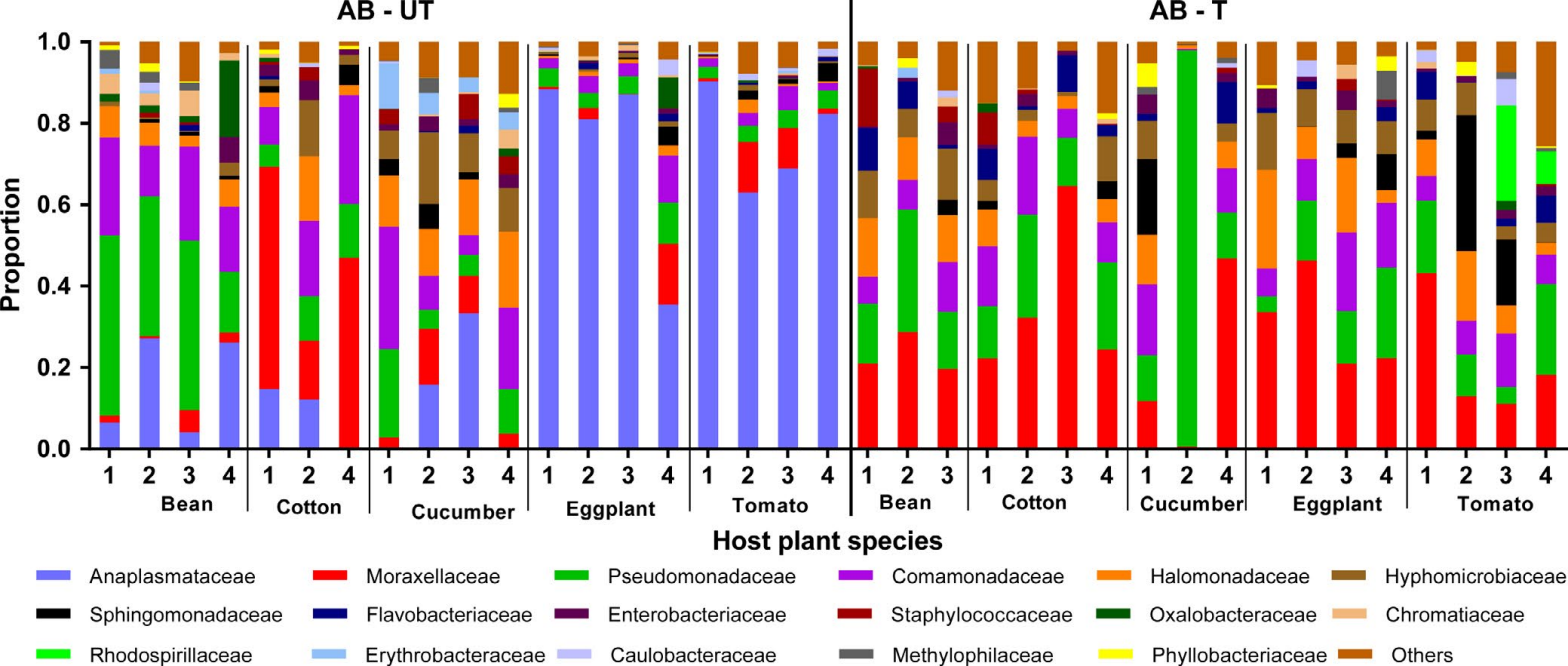
Family‐level bacterial composition of antibiotic‐untreated and antibiotic‐treated spider mites that were reared for six generations on different host plants, as deduced by massive 16S rRNA sequencing

Diversity and species richness index values are provided in Supporting Information Table S1, and their mean (±*SEM*) is plotted in Figure 2. OTU identification resulted in 54 genera distributed in 36 families, 25 orders, and seven phyla of bacteria (Figure 3; Supporting Information Figure S2). Good's coverage for each sample was more than 99% (Supporting Information Table S1). Overall most of the sequences from the bacterial communities associated with *T. truncatus* lines belonged to Gammaproteobacteria (44.95%), followed by Alphaproteobacteria (33.91%), Betaproteobacteria (13.54%), and Flavobacteria (2.22%) (Supporting Information Table S2).

### 3.2 Changes in bacterial communities with host plants and antibiotic treatment

**Figure 4:**
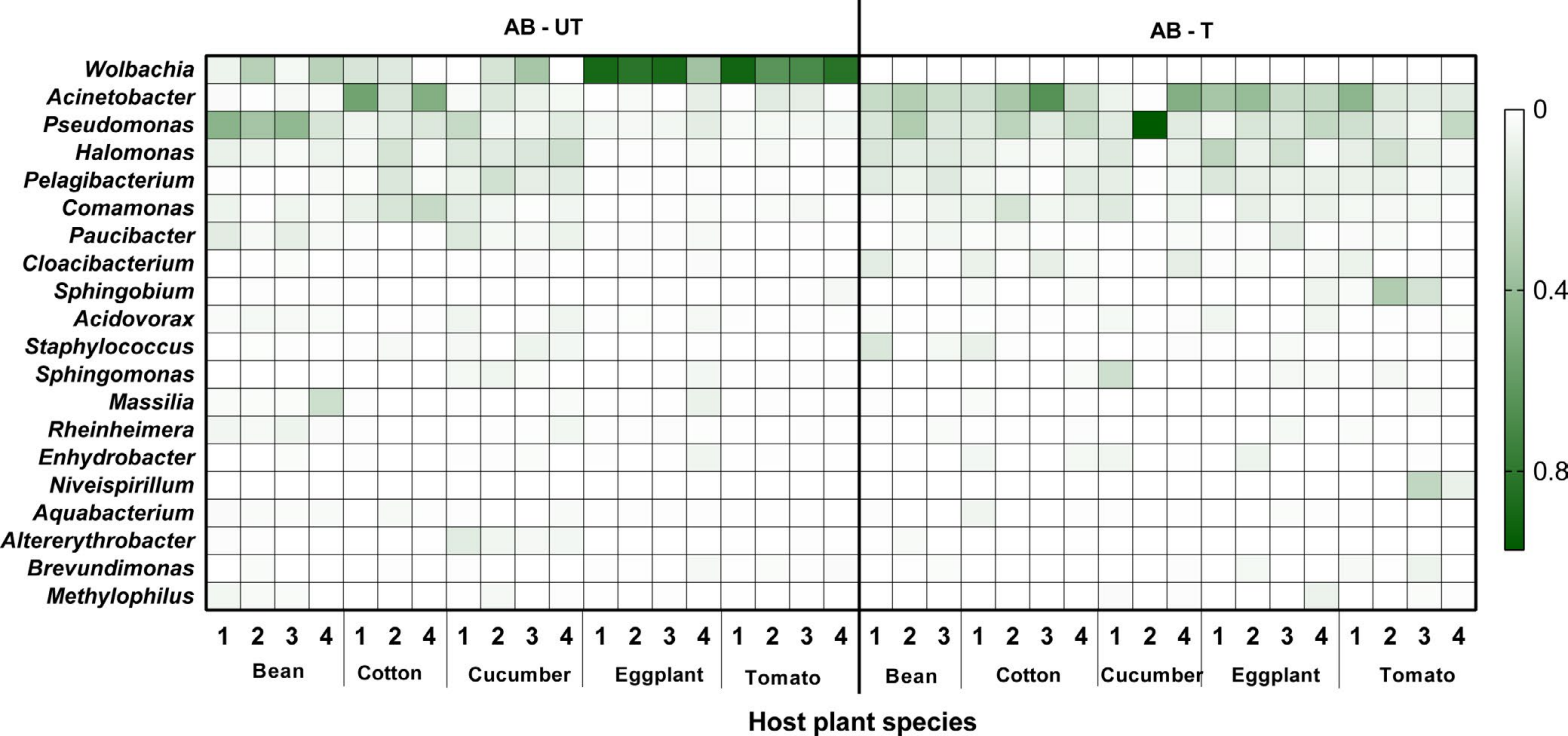
Heatmap of relative abundance for the 20 most abundant OTUs from spider mites that were reared for six generations on different host plants

The species diversity of spider mite bacterial communities, indicated by Shannon indexes, was significantly affected by the interaction between host plant species and antibiotics (*F_4,27_* = 4.80, *p* < 0.01). The significant interactions found for Shannon indexes come from a significant effect of antibiotic treatment in eggplant (*t = *3.20, *p* = 0.02) and tomato (*t = *3.40, *p* = 0.01), while it has no effect on the other three plant species (bean: *t = *0.28, *p* = 0.99; cotton: *t = *0.39, *p* = 0.99; cucumber: *t = *1.91, *p* = 0.29; Figure 2). The number of observed OTUs was significantly affected by host plant species (*F_4,31_* = 2.68, *p* < 0.05) and antibiotics (*F_1,31_* = 13.16, *p* < 0.01), but not by their interaction (*F_4,27_* = 1.89, *p* = 0.14). Thus, except for spider mites reared on cotton, OTU number richness index was reduced in lines that had been exposed to antibiotics when reared on the same plant species, which suggest that the species richness of bacteria tended to decrease after antibiotic exposure (Figure 2). Moreover, there were substantial plant‐specific variations of bacterial composition at the family (Figure 3) and phylum levels (Supporting Information Figure S2), and in the relative abundance of the 10 most common OTUs (Supporting Information Table S2; Figure 4) between treated and untreated lines. Antibiotic treatment significantly increased the relative abundances of OTU4 (*Acinetobacter*) (*F_1,31_* = 9.65, *p* < 0.05) and OTU10 (*Cloacibacterium*) (*F_1,31_* = 10.36, *p* < 0.05) in spider mites on five host plants; however, the difference among them was not significant (*F_4,31_* = 2.06, *p* = 0.11; *F_4,31_* = 0.67, *p* = 0.62, respectively; Supporting Information Figure S3). Overall, these results indicated that the species richness and diversity of spider mites bacterial communities depend on host plant species and antibiotics.

**Figure 5:**
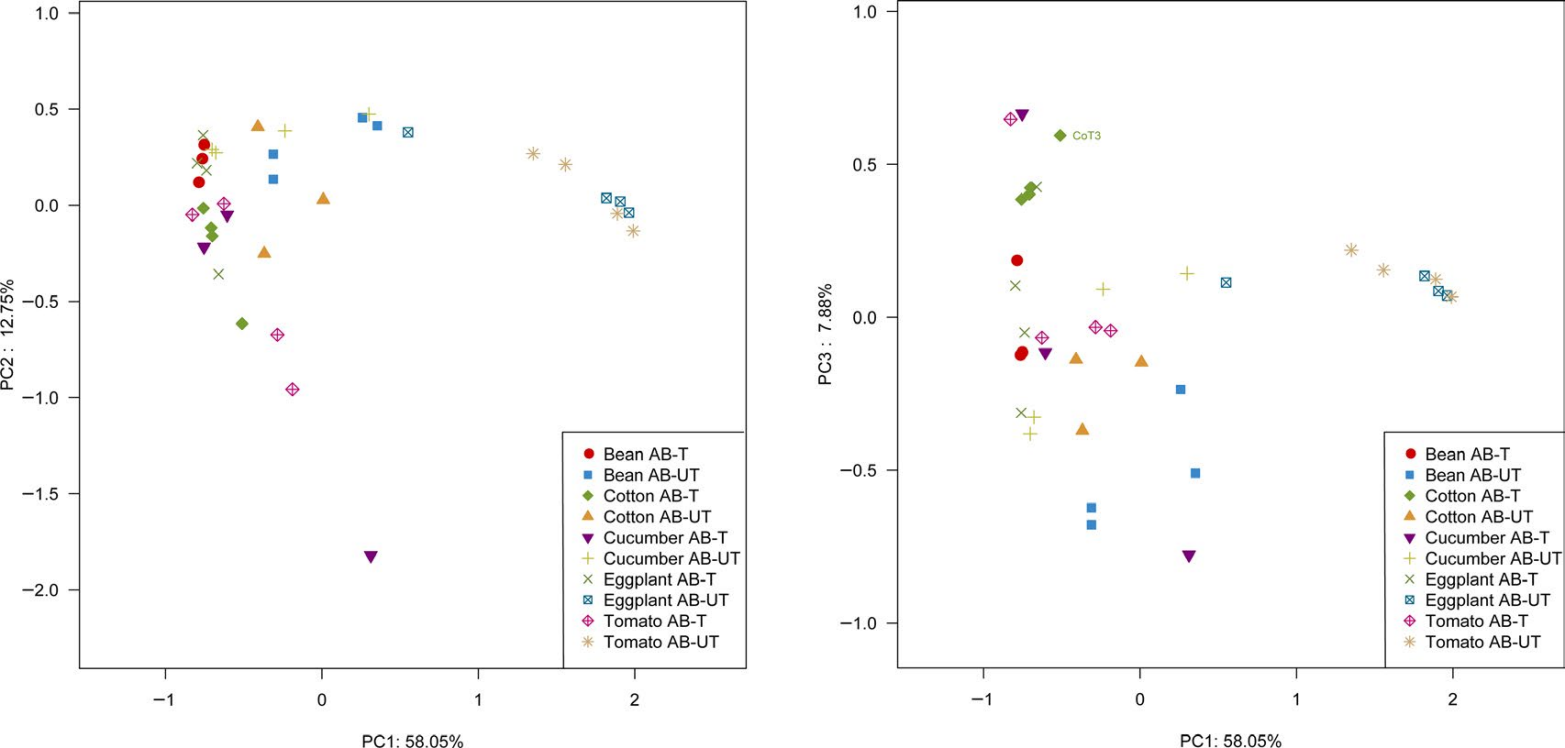
Principal coordinates analysis (PCoA) comparing bacterial communities of antibiotic‐untreated and antibiotic‐treated spider mites that were reared on different host plants. PCoA based on Bray–Curtis distance values computed for whole communities from spider mites that were reared on different host plants

To visualize variation in bacterial community structure on different samples, we plotted the results of a PCoA based on Bray–Curtis dissimilarity distances (Figure 5). These distances and weighted UniFrac distances revealed significant differences in bacterial communities when mite lines were maintained on the different plant species (Bray–Curtis: *F_9,36_ = *4.422, *R* ^2^ = 0.596, *p < *0.001; weighted UniFrac: *F_9,36_* = 6.237, *R* ^2^ = 0.675, *p < *0.001). MRPP analyses also revealed that bacterial communities were significantly different among host types (*p < *0.001).

### 3.3 Abundance of *Wolbachia* and *Spiroplasma* on different plants

**Figure 6:**
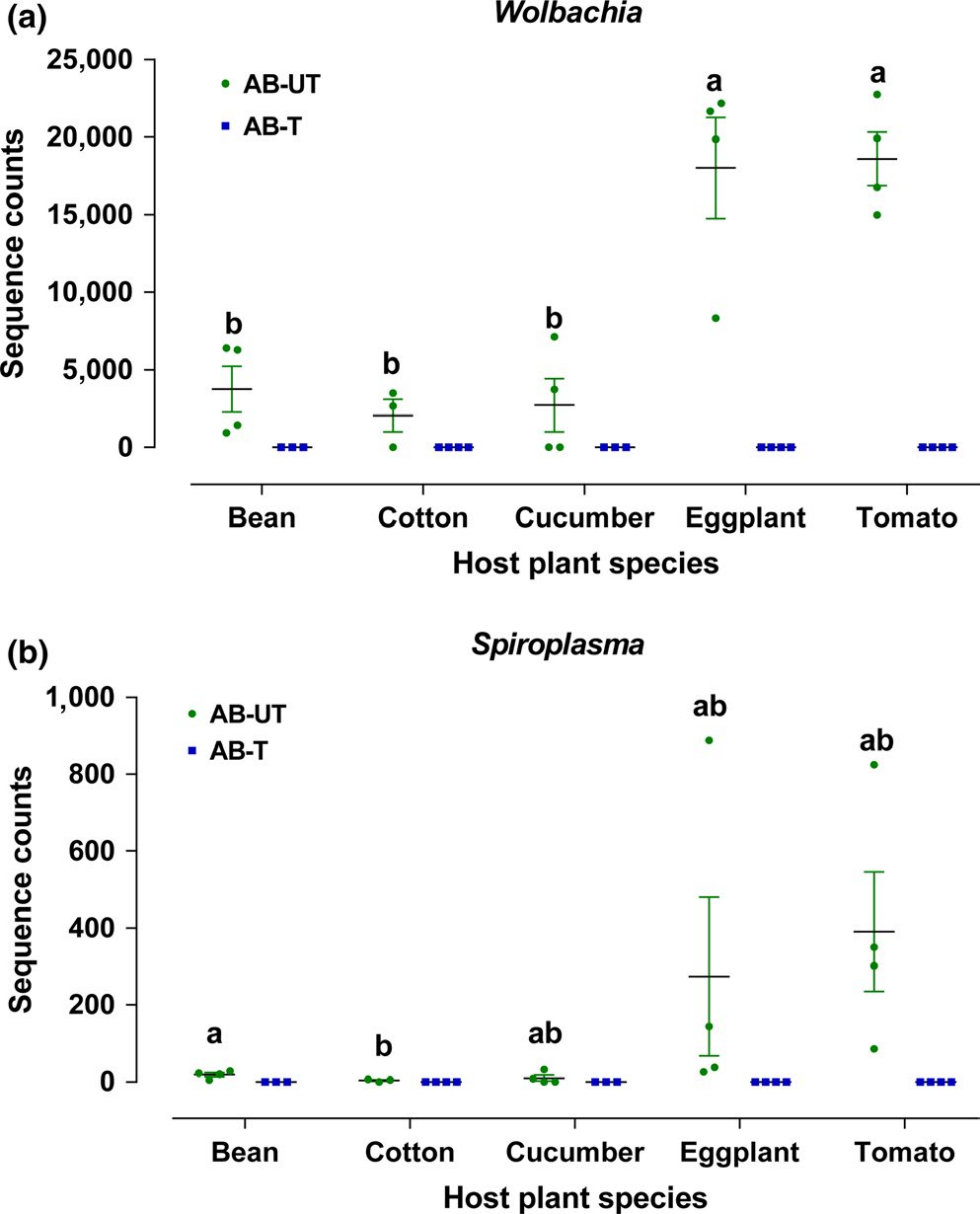
Sequence counts of two OTUs of antibiotic‐untreated and antibiotic‐treated spider mites that were reared for six generations on different host plants. (a) *Wolbachia*; (b) *Spiroplasma*. Horizontal lines indicate the mean of biological replicates. Superscripts (a, b) above horizontal lines indicate significant differences between host plants (*p* < 0.05)

The facultative endosymbionts *Wolbachia* and *Spiroplasma* were not detected in any of the spider mites reared on any of the plants in populations treated with antibiotics (Supporting Information Figure S4; Figure 6). There were significant effects of host plant species on the relative abundances of *Wolbachia* (LR Chisq = 52.89, *df* = 4, *p* < 0.001) and *Spiroplasma* (LR Chisq = 52.89, *df* = 4, *p* < 0.001) in untreated mites (Figure 6). *Wolbachia* and *Spiroplasma* were more abundant in spider mites that fed on tomato and eggplant compared with all other host plants (Supporting Information Figure S4; Figure 6).

### 3.4 Endosymbiont effects on *T. truncatus* performance

**Figure 7:**
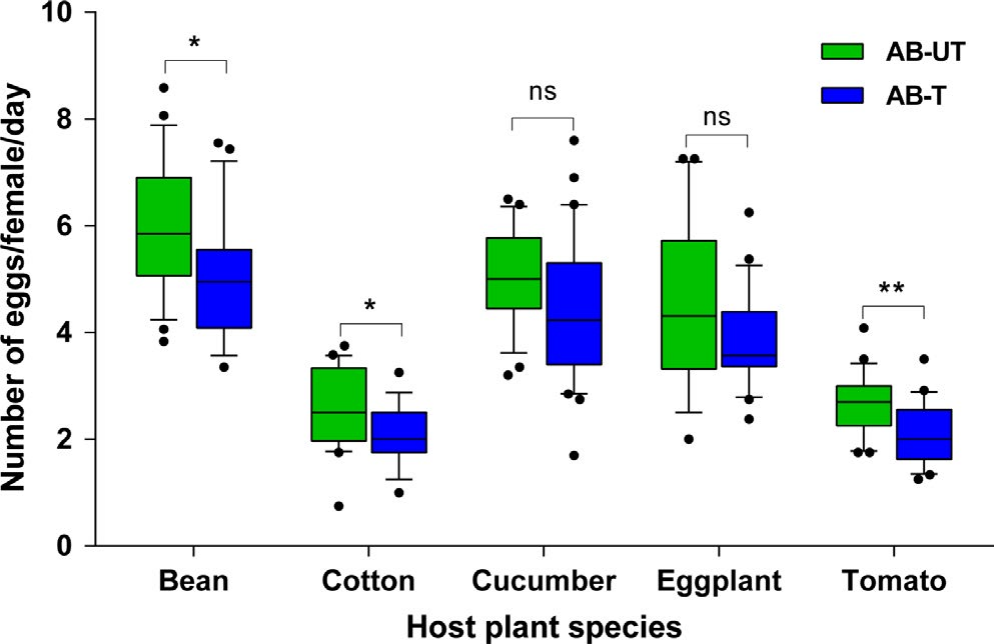
Fecundity (number of eggs laid/female/day) of spider mites on different plant species. Horizontal lines in the boxes represent medians, whiskers represent the 10th–90th percentiles, and dots represent data points outside of this range. “*” represents significant difference between antibiotic‐untreated and antibiotic‐treated spider mites on the same host plant (*p* < 0.05)

The average number of eggs laid per female per day was significantly affected by antibiotic (*F_1,224_* = 20.04; *p* < 0.001) and host plants (*F_4,224_* = 84.59; *p* < 0.001), but not by their interaction (*F_4,220_* = 20.04; *p* = 0.879). Both of AB‐T and AB‐UT spider mites that fed on bean, cucumber, and eggplant laid more eggs compared with mites fed on cotton and tomato (Figure 7). Antibiotic treatment significantly reduced fecundity on bean (*t* = −2.54; *p* < 0.05), cotton (*t* = −2.34; *p* < 0.05), and tomato (*t* = −3.41; *p* < 0.01) (Figure 7).

## 4 DISCUSSION

In this study, we investigated the impact of host plant and antibiotic treatment on bacterial diversity and bacterial community composition of the spider mite *T. truncatus*. We demonstrated that the bacterial diversity of *T. truncatus* was influenced by host plant species and antibiotic. In particular, the abundance of the facultative endosymbionts *Wolbachia* and *Spiroplasma* was also influenced by host plant species and was completely eliminated by the antibiotic treatment. Intriguingly, when assessing offspring production in the mites exposed to the different conditions, we found that daily fecundity tended to be lower in the mites with reduced bacterial diversity following the antibiotic treatment across five host plants. The results highlight that host plants and antibiotics can shape spider mite bacterial communities and that bacterial symbionts improve mite performance.

### 4.1 Host plant and antibiotic treatment effects on the spider mite microbial communities

In this study, the host plants tested strongly altered the composition of the microbial community (diversity and abundance) in spider mites. Similarly, research on Colorado potato beetles (Chung et al., 2017), whiteflies (Su et al., 2016), aphids (Guidolin & Cônsoli, 2016), and pine processionary moths (Strano et al., 2017) have shown that populations that fed on different plant species had differing microbial communities. Here, we found that spider mites that were switched from bean to other host plants experienced changes in bacterial community diversity and species richness, especially for the main bacterial species. These changes could reflect immediate effects of host plants on bacterial communities after mites fed on the plants, or longer term effects given that mites were held on different host plants for multiple generations. The changes observed here may reflect the role of the microbial community in the ability of spider mites to cope with different host plants (Hansen & Moran, 2014; Jaenike, 2015; Tsuchida, Koga, & Fukatsu, 2004), but testing such hypothesis is beyond the scope of this study (i.e., fecundity was only characterized on the host plants on which the lines were maintained).

The relative abundance of maternally inherited endosymbionts should be taken into account when studying bacterial communities across host populations, as variation in bacterial density may impact host biology and ecology (Fromont, Riegler, & Cook, 2017). In the current study, both *Wolbachia* and *Spiroplasma* were detected in *T. truncatus*, which were also widespread in *T. urticae* (Enigl & Schausberger, 2007; Hoy & Jeyaprakash, 2005). We also found that host plant had effects on the relative amounts of *Wolbachia* and *Spiroplasma*. Changes in relative abundance of symbionts with host have been noted for other polyphagous insects, including pea aphids (Tsuchida et al., 2002), cowpea aphid (Brady & White, 2013), chestnut weevils (Toju & Fukatsu, 2011), and others (Pan et al., 2014). There are several potential explanations for the effects of host plant species on symbiotic bacterial communities. First, plant secondary metabolites or phytotoxins may possess variable antibacterial activities (Harborne, 1993), which could influence population growth (Kohl & Dearing, 2012). Each plant has a different secondary metabolite profile, such as terpenoids and glycoalkaloids in tomato (Falara et al., 2011; Milner et al., 2011), glycoalkaloids in eggplant (Milner et al., 2011), cucurbitacin in cucumber (Balkema‐Boomstra et al., 2003), protease inhibitors in bean (Visôtto, Oliveira, Ribon, Mares‐Guia, & Guedes, 2009), and gossypol in cotton (Bottger, Sheehan, & Lukefahr, 1964). In addition to secondary metabolites, physical properties of different host plants, such as leaf toughness and trichomes, may influence bacterial communities, because they directly impact spider mite growth and physiochemical interactions between bacteria and their insect hosts (Chung et al., 2017). Alternatively, the host may manipulate its endosymbiont titer to compensate for specific deficiencies in the nutrient profile of its host plant (Zhang, Cao, et al., 2016). However, at this stage, it is unclear whether the high relative abundances of *Wolbachia* and *Spiroplasma* in mite lines from tomato and eggplant are adaptively significant.

Previous studies have shown that antibiotic treatment alters the bacterial community of mites and other herbivores; in particular, antibiotic treatment influences the relative abundance of some facultative endosymbionts (Breeuwer, 1997; Lehman et al., 2009; Staudacher et al., 2017). Here, the number of OTUs and species diversity were reduced regardless of host plant. The facultative endosymbionts *Wolbachia* and *Spiroplasma* were completely absent from the antibiotic‐treated populations, which were consistent with the effects produced by antibiotics in previous studies (Staudacher et al., 2017; Xie, Sun, Xue, & Hong, 2016).

One unexpected finding was that the relative abundance of *Acinetobacter*,* Pseudomonas*,* Halomonas*,* Pelagibacterium,* and *Cloacibacterium* was increased by antibiotic treatment. Prior exposure to plant toxins can enhance the diversity of gut microbes in herbivores (Kohl & Dearing, 2012). Bacterial taxa frequently reported in plants include the genera *Pseudomonas*,* Bradyrhizobium*,* Azorhizobium*,* Azospirillum,* and *Bacillus* (Partida‐Martinez & Heil, 2011). Perhaps, *Pseudomonas* and *Bacillus,* which were detected in *T. truncatus* in this study, may have been obtained from the host plant. Antibiotic treatments that remove part of the microbial community might then lead to a suitable living environment for other bacteria. This might correspond to de novo colonization after treatment. It has to be noted that, the symbiotic bacterial communities of arthropod herbivores may also be affected by host genotype (Brady et al., 2014), how the host impact the plant–bacterial community interaction in spider mite should thus be considered in future studies.

### 4.2 Host plant and antibiotic treatment effects on *T. truncatus* performance

Many facultative endosymbionts are not essential for host survival but can have an important impact on insect life history traits (Giron et al., 2017; O'Neill, Werren, & Hoffmann, 1997). Our previous study showed that reproductive parasites, such as *Wolbachia*,* Cardinium,* and *Spiroplasma*, are widespread in *T. truncatus* (Zhang, Chen, et al., 2016), and those facultative endosymbionts influence host reproduction via various phenotypic effects (Engelstädter & Hurst, 2009). The abundance of *Wolbachia* and *Spiroplasma* in spider mites was strongly influenced by host plant, and the presence of these endosymbionts can enhance spider mite performance in a host plant‐specific manner. Moreover, the presence of these endosymbionts is positively correlated with spider mite fecundity on specific plants.

A couple of different scenarios might explain how these endosymbionts can influence spider mite performance on different host plant species. Herbivore‐associated microbes can positively and negatively influence insect fitness by mediating plant defenses and detoxifying phytochemicals (Chung et al., 2013, 2017 ) or enzymes, such as hydrolases, glucosidases, phosphatases, and glutathione transferases (Dowd & Shen, 1990; Shen & Dowd, 1991). For instance, the presence/absence of the bacterial endosymbionts *Wolbachia*,* Cardinium,* or/and *Spiroplasma* in the spider mite *T. urticae* has also been previously reported as altering distinct plant defense parameters and affecting mite performance, but there were no indications of a causal link between the two (Staudacher et al., 2017). Western corn rootworms (*Diabrotica virgifera*) infected with *Wolbachia* suppressed defense‐related genes in maize roots and altered host performance compared with uninfected rootworms (Barr, Hearne, Briesacher, Clark, & Davis, 2010). Another explanation is that microbes could improve the nutritional properties of the herbivore's diet for certain plant species, which allows females to allocate more resources to egg production (Douglas, 1998; Feldhaar et al., 2007). *Buchnera* synthesize essential amino acids and other substances that are absent from their host aphid's diet, and if the symbiont is removed, the host grows very slowly and cannot reproduce (Douglas, 1996; Koga, Tsuchida, & Fukatsu, 2003). *Wolbachia* genome analysis revealed that they lack many essential biosynthetic pathways (Wu et al., 2004). Therefore, it is likely that *Wolbachia* impose a nutritional burden on their hosts, and host–symbiont competition for key resources, such as amino acids (Caragata, Rances, O'Neill, & McGraw, 2014), sugars (Markov & Zakharov, 2006), or iron (Gill, Darby, & Makepeace, 2014), could influence host performance.

Although the antibiotic treatments resulted in the complete removal of *Wolbachia* and *Spiroplasma* from the mites, various other bacterial strains were also affected, making attribution to specific bacteria difficult. Future experiments might focus on the performance of spider mites with or without symbionts from natural populations with multiple host plants. Polyphagous arthropod‐associated microbes could have played a role in adaptation to new hosts and host range expansion (Chu, Spencer, Curzi, Zavala, & Seufferheld, 2013; Jaenike, 2015). Because *T. truncatus* is a polyphagous pest and harbors different species of bacterial symbionts, an association of reproductive bacteria with spider mites might help them adapt to new host plants.

Host plant‐dependent impacts of symbiotic microorganisms on the fitness of herbivorous insects may be a widespread and currently unrecognized dimension in insect–plant interactions (Chandler et al., 2008). Here, we found that host plant and antibiotic treatment influenced the *T. truncatus* symbiotic bacterial community; in particular, there were effects on the relative abundances of the endosymbionts *Wolbachia* and *Spiroplasma* in *T. truncatus*, which in turn may have altered host performance. Further studies are needed to determine whether spider mite‐associated microbes alter spider mite fitness by mediating plant defenses and detoxifying phytochemicals. Documenting the presence of the microbial community and identifying their effects on hosts can have important implications for the management of this pest species (Crotti et al., 2012; Oliver, Degnan, Burke, & Moran, 2010).

## CONFLICT OF INTERESTS

The authors state that there are no conflict of interests.

## AUTHORS CONTRIBUTION

YXZ and XYH conceived and designed the experiments; YXZ and YLS performed the experiments; YXZ and PYJ analyzed the data with guidance from AAH; YXZ, AAH, and XYH wrote the manuscript. All authors discussed the results and commented on the manuscript.

## ETHICS STATEMENT

No specific permissions were required for the collection because *T. truncatus* is a pest on the bean*. Tetranychus truncatus* is not an endangered species in China and is not protected by law. No ethical approval was required to work with this species in our study.

## Data Availability

Illumina sequence reads are available on the NCBI Sequence Read Archive (SRA) under accession number SRP158961.
