# Supplementary material for: A change in the bacterial community of spider mites decreases fecundity on multiple host plants
Source: Microbiologyopen. 2018 Oct 11;8(6):e00743. doi: 10.1002/mbo3.743 (PMC6562136; doi:10.1002/mbo3.743)
Supplement: Supplementary file 1 [file MBO3-8-e00743-s001.pdf]

SUPPORTING INFORMATION

Article title: A change in the bacterial community of spider mites decreases fecundity on multiple host plants

Authors: Yu-Xi Zhu[1], Yue-Ling Song[1], Ary A. Hoffmann[2], Peng-Yu Jin[1], Shi-Mei Huo[1] and Xiao-Yue Hong[1]

[1]Department of Entomology, Nanjing Agricultural University, Nanjing, Jiangsu 210095, China

[2] School of BioSciences, Bio21 Institute, The University of Melbourne, Victoria 3010, Australia

Author for correspondence

Xiao-Yue Hong

Department of Entomology, Nanjing Agricultural University, Nanjing, Jiangsu 210095, China

E-mail: xyhong@njau.edu.cn

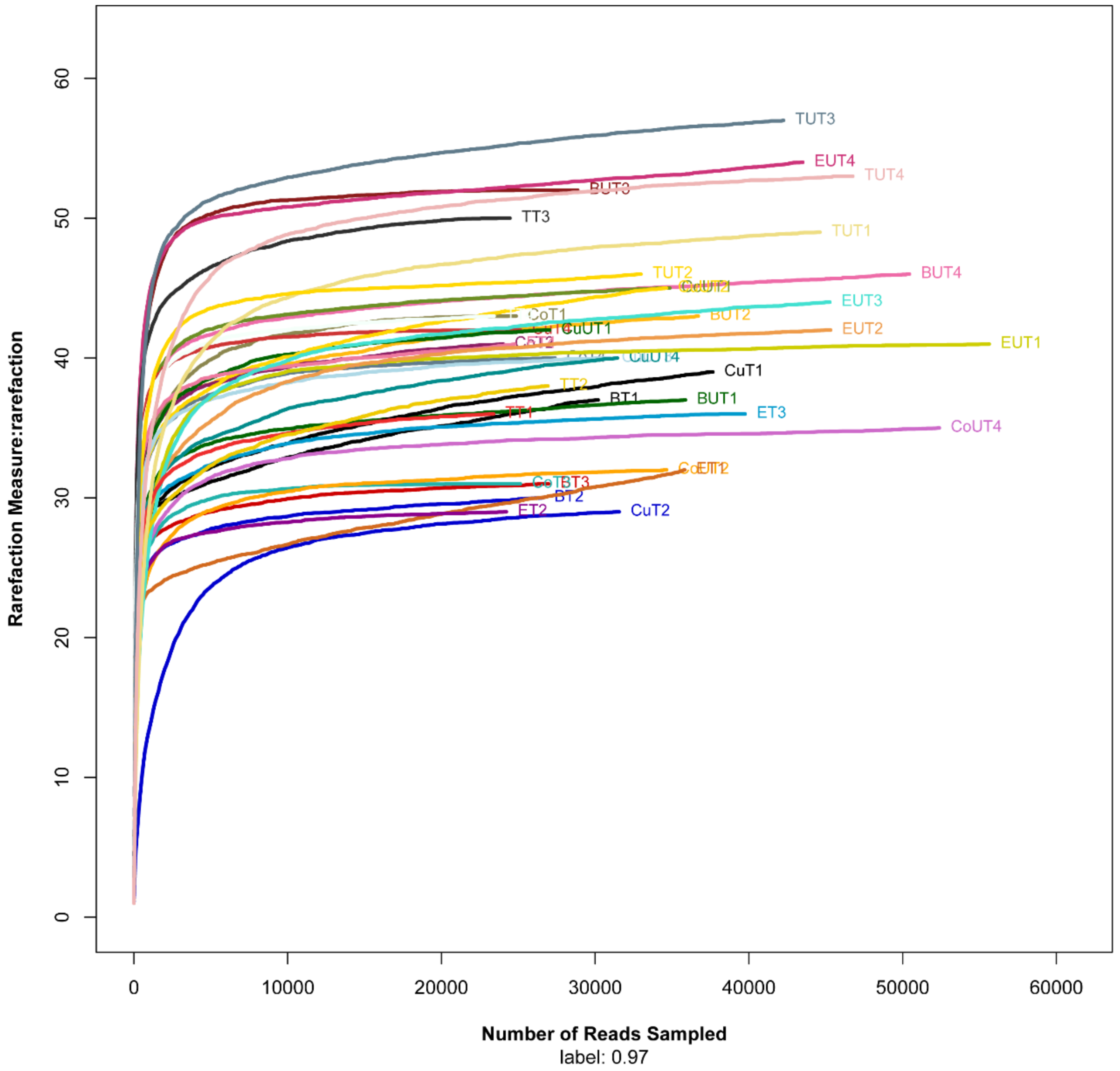

FIGURE S1 Rarefaction curves for 16S bacterial communities of spider mites that were reared for 6 generations on different host plants.

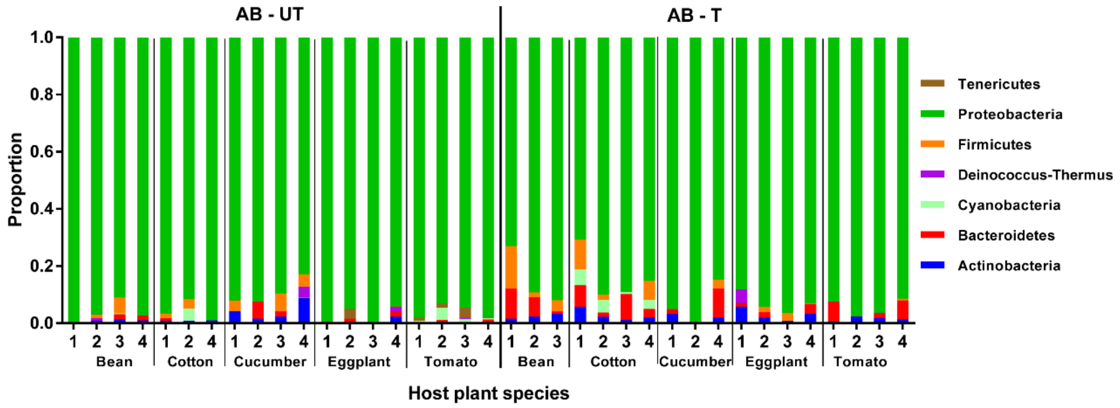

FIGURE S2 Relative abundance of bacterial communities at the phylum level in spider mites that were reared for 6 generations on different host plants.

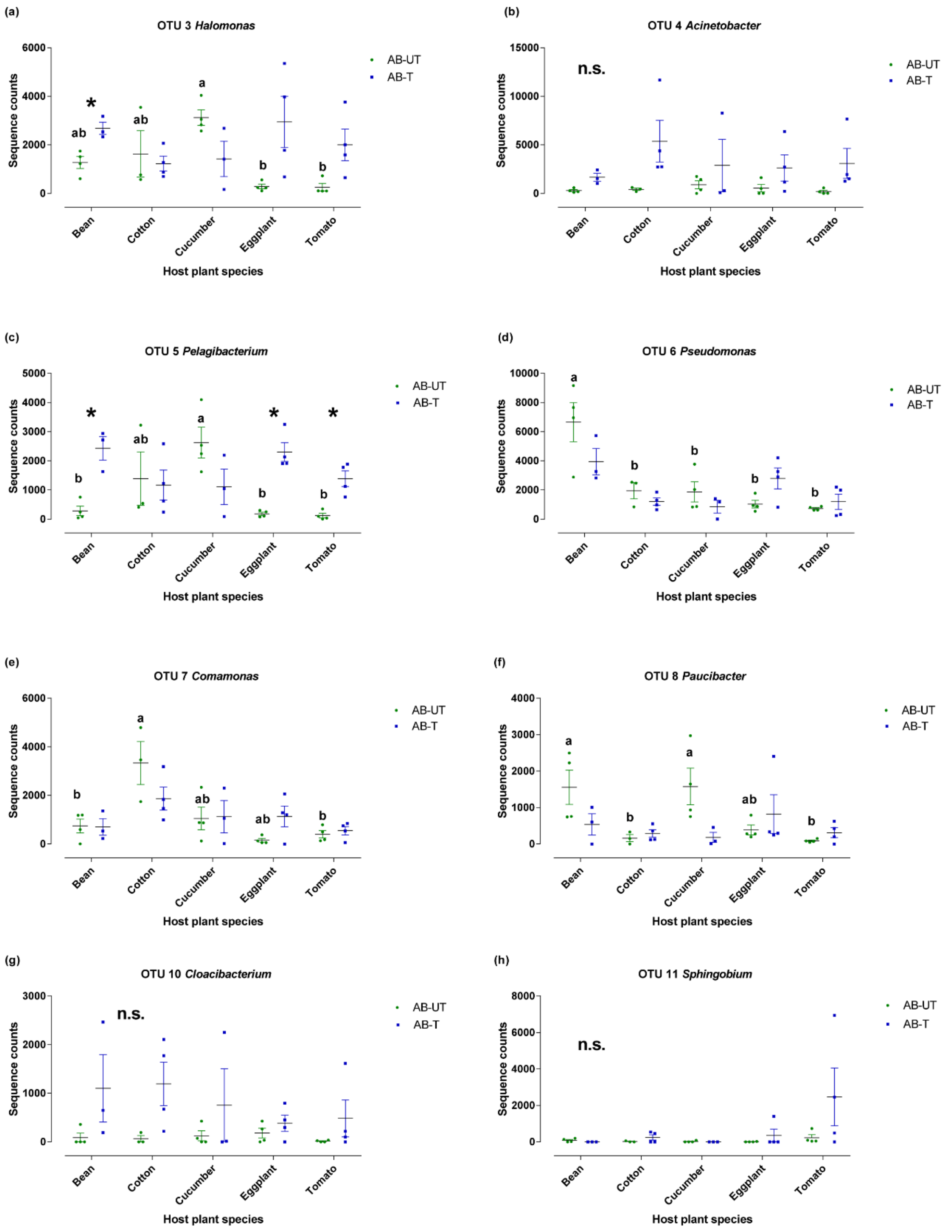

FIGURE S3 Sequence counts of eight OTUs of antibiotic-untreated and -treated spider mites that were reared for 6 generations on different host plants. (a) *Halomonas*; (b) *Acinetobacter*; (c) *Pelagibacterium*; (d) *Pseudomonas*; (e) *Comamonas*; (f) *Paucibacter*; (g) *Cloacibacterium*; (h) *Sphingobium*. Superscripts (a, b) above horizontal lines indicate significant differences between antibiotic-untreated mites that were reared on different host plants (P < 0.05). "*" represent significant different between antibiotic-untreated and -treated spider mites on the same host plant (P < 0.05). n.s.: not significant.

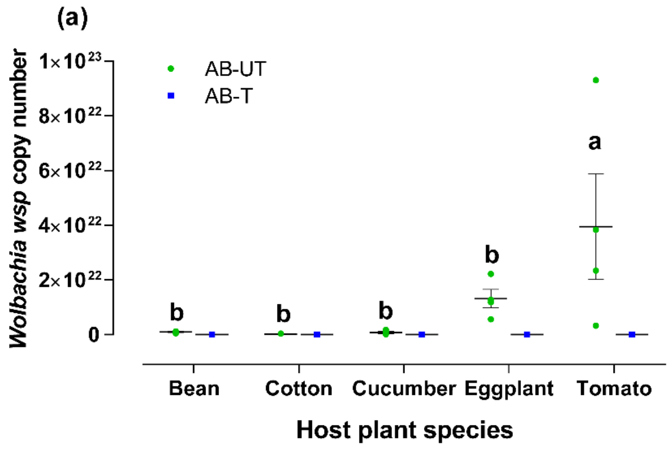

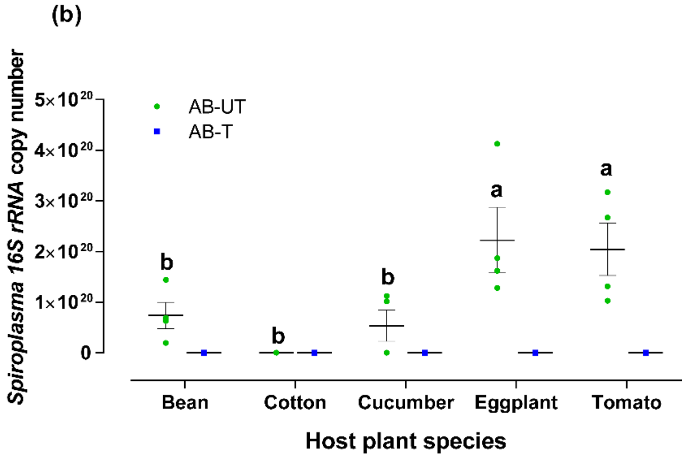

FIGURE S4 Density of two endosymbionts in antibiotic-untreated and -treated spider mites that were reared for 6 generations on different host plants. (a) *Wolbachia* ; (b) *Spiroplasma*. Horizontal lines indicate the mean of biological replicates. Superscripts (a, b) above horizontal lines indicate significant differences between host plants ($P < 0.05$).

TABLE S1 Alpha diversity indexes from spider mite that were reared for 6 generation on different host plants.

Host- plant species	Antibiotic treatment	Replicate	Ace	Chao	Shannon	Simpson	Coverage	OTUs
**Bean**	**AB-T**	1	41.82	38.50	2.67	0.09	0.9999	35.00
		2	31.96	31.00	2.56	0.12	0.9999	30.00
		3	31.44	31.00	2.85	0.07	1.0000	31.00
		**Mean ± SEM**	**35.07±3.38**	**33.50±2.50**	**2.69±0.08**	**0.09±0.01**	**0.9999**	**32.00±1.53**
	**AB-UT**	1	38.64	38.00	2.36	0.18	0.9999	37.00
		2	44.95	43.33	2.43	0.17	0.9999	40.00
		3	52.00	52.00	2.73	0.13	1.0000	52.00
		4	55.75	49.00	2.78	0.10	0.9999	44.00
		**Mean ± SEM**	**47.83±3.80**	**45.58±3.10**	**2.57±0.11**	**0.15±0.02**	**1.0000**	**43.25±3.25**
**Cotton**	**AB-T**	1	43.00	43.00	3.06	0.06	1.0000	43.00
		2	42.44	41.50	2.76	0.10	0.9999	41.00
		3	31.00	31.00	2.03	0.27	1.0000	31.00
		4	40.42	40.00	2.89	0.08	1.0000	40.00
		**Mean ± SEM**	**39.21±2.79**	**38.88±2.70**	**2.69±0.23**	**0.13±0.05**	**1.0000**	**38.75±2.66**
	**AB-UT**	1	46.36	45.00	2.71	0.10	1.0000	45.00
		2	32.46	32.00	2.48	0.11	1.0000	31.00
		4	38.49	36.00	2.37	0.14	1.0000	34.00
		**Mean ± SEM**	**39.10±4.02**	**37.67±3.84**	**2.52±0.10**	**0.12±0.01**	**1.0000**	**36.67±4.26**
**Cucumber**	**AB-T**	1	48.47	44.00	2.79	0.08	0.9999	37.00
		2	29.80	29.50	0.37	0.86	0.9999	29.00
		4	42.00	42.00	2.64	0.15	1.0000	42.00
		**Mean ± SEM**	**40.09±5.47**	**38.50±4.54**	**1.94±0.78**	**0.37±0.25**	**0.9999**	**36.00±3.79**
	**AB-UT**	1	43.13	42.50	2.72	0.09	0.9999	41.00
		2	60.63	48.33	2.79	0.09	0.9999	44.00

		3	40.95	40.00	2.55	0.14	1.0000	40.00
		4	43.84	43.00	2.93	0.07	0.9999	38.00
		**Mean ± SEM**	**47.14±4.54**	**43.46±1.75**	**2.75±0.08**	**0.10±0.01**	**0.9999**	**40.75±1.25**
**Eggplant**	**AB-T**	1	156.91	53.00	2.29	0.16	0.9998	28.00
		2	29.59	29.00	2.68	0.08	1.0000	29.00
		3	36.57	36.00	2.80	0.08	1.0000	34.00
		4	46.51	41.50	2.97	0.08	0.9999	40.00
		**Mean ± SEM**	**67.40±30.04**	**39.88±5.07**	**2.68±0.14**	**0.10±0.02**	**0.9999**	**32.75±2.75**
	**AB-UT**	1	41.42	41.00	0.81	0.74	1.0000	40.00
		2	42.79	43.00	1.22	0.60	1.0000	41.00
		3	46.38	47.00	0.90	0.70	0.9999	44.00
		4	105.65	60.00	2.77	0.14	0.9999	51.00
		**Mean ± SEM**	**59.06±15.57**	**47.75±4.27**	**1.42±0.46**	**0.55±0.14**	**0.9999**	**44.00±2.48**
**Tomato**	**AB-T**	1	36.87	36.00	2.55	0.14	1.0000	36.00
		2	42.75	41.00	2.44	0.14	0.9999	38.00
		3	50.00	50.00	2.94	0.09	1.0000	50.00
		4	43.64	43.00	2.88	0.08	1.0000	43.00
		**Mean ± SEM**	**43.32±2.69**	**42.50±2.90**	**2.70±0.12**	**0.11±0.02**	**0.9999**	**41.75±3.12**
	**AB-UT**	1	51.11	50.50	0.74	0.77	0.9999	47.00
		2	54.89	47.00	1.88	0.38	0.9999	45.00
		3	62.09	58.00	1.72	0.45	0.9999	55.00
		4	54.48	53.33	1.11	0.64	1.0000	52.00
		**Mean ± SEM**	**55.64±2.31**	**52.21±2.32**	**1.36±0.27**	**0.56±0.09**	**0.9999**	**49.75±2.29**

TABLE S2 Relative abundance of the 10 most abundant OTUs from spider mites that were reared on different host plants.

Host- plant species	Antibiotic treatment	Replicate	*Wolbachia*	*Acinetobacter*	*Pseudomonas*	*Halomonas*	*Pelagibacterium*	*Comamonas*	*Paucibacter*	*Cloacibacterium*	*Sphingobium*	*Acidovorax*
**Bean**	**AB-T**	1	0.00	0.21	0.15	0.14	0.12	0.01	0.00	0.11	0.00	0.02
		2	0.00	0.29	0.30	0.10	0.07	0.02	0.03	0.03	0.00	0.00
		3	0.00	0.20	0.14	0.11	0.13	0.06	0.04	0.01	0.00	0.00
		**Mean ± SEM**	0.00±0.00	0.23±0.03	0.20±0.05	0.12±0.01	0.11±0.02	0.03±0.02	0.02±0.01	0.05±0.03	0.00±0.00	0.01±0.01
	**AB-UT**	1	0.06	0.02	0.44	0.08	0.01	0.06	0.11	0.00	0.00	0.02
		2	0.27	0.01	0.34	0.06	0.00	0.00	0.03	0.00	0.01	0.04
		3	0.04	0.04	0.42	0.03	0.00	0.06	0.10	0.02	0.00	0.03
		4	0.26	0.02	0.15	0.07	0.03	0.03	0.03	0.00	0.01	0.02
		**Mean ± SEM**	0.16±0.06	0.02±0.01	0.34±0.07	0.06±0.01	0.01±0.01	0.04±0.01	0.07±0.02	0.01±0.01	0.01±0.00	0.03±0.00
**Cotton**	**AB-T**	1	0.00	0.18	0.13	0.09	0.05	0.07	0.02	0.08	0.02	0.01
		2	0.00	0.32	0.25	0.04	0.03	0.16	0.02	0.01	0.00	0.01
		3	0.00	0.65	0.12	0.03	0.01	0.05	0.01	0.09	0.00	0.01
		4	0.00	0.20	0.21	0.06	0.11	0.08	0.01	0.03	0.02	0.00
		**Mean ± SEM**	0.00±0.00	0.34±0.11	0.18±0.03	0.06±0.01	0.05±0.02	0.09±0.02	0.02±0.00	0.05±0.02	0.01±0.01	0.01±0.00
	**AB-UT**	1	0.15	0.54	0.06	0.03	0.02	0.08	0.01	0.01	0.00	0.00
		2	0.12	0.14	0.11	0.16	0.14	0.15	0.00	0.00	0.00	0.00
		4	0.00	0.47	0.13	0.03	0.02	0.21	0.01	0.00	0.00	0.01
		**Mean ± SEM**	0.09±0.05	0.38±0.12	0.10±0.02	0.07±0.04	0.06±0.04	0.15±0.04	0.01±0.00	0.00±0.00	0.00±0.00	0.00±0.00
**Cucumber**	**AB-T**	1	0.00	0.07	0.11	0.12	0.09	0.12	0.00	0.00	0.00	0.04
		2	0.00	0.01	0.97	0.01	0.00	0.00	0.00	0.00	0.00	0.00
		4	0.00	0.47	0.11	0.06	0.04	0.07	0.02	0.10	0.00	0.01
		**Mean ± SEM**	0.00±0.00	0.18±0.14	0.40±0.29	0.06±0.03	0.04±0.03	0.06±0.03	0.01±0.01	0.03±0.03	0.00±0.00	0.02±0.01
	**AB-UT**	1	0.00	0.02	0.22	0.13	0.07	0.11	0.13	0.00	0.00	0.05
		2	0.16	0.13	0.05	0.12	0.18	0.04	0.04	0.00	0.00	0.00

		3	0.33	0.07	0.05	0.14	0.10	0.01	0.03	0.02	0.00	0.01
		4	0.00	0.04	0.11	0.19	0.11	0.05	0.07	0.00	0.00	0.05
		**Mean ± SEM**	0.12±0.08	0.07±0.02	0.11±0.04	0.15±0.02	0.12±0.02	0.05±0.02	0.07±0.02	0.01±0.01	0.00±0.00	0.03±0.01
**Eggplant**	**AB-T**	1	0.00	0.34	0.04	0.24	0.14	0.00	0.01	0.01	0.00	0.05
		2	0.00	0.39	0.15	0.08	0.09	0.09	0.01	0.02	0.00	0.00
		3	0.00	0.21	0.13	0.18	0.08	0.05	0.10	0.00	0.00	0.00
		4	0.00	0.22	0.22	0.03	0.08	0.07	0.01	0.03	0.06	0.05
		**Mean ± SEM**	0.00±0.00	0.29±0.04	0.14±0.04	0.13±0.05	0.10±0.01	0.05±0.02	0.03±0.02	0.02±0.01	0.02±0.02	0.03±0.01
	**AB-UT**	1	0.88	0.00	0.05	0.01	0.00	0.00	0.01	0.00	0.00	0.01
		2	0.81	0.03	0.04	0.01	0.00	0.00	0.01	0.01	0.00	0.01
		3	0.87	0.00	0.04	0.01	0.01	0.01	0.01	0.00	0.00	0.00
		4	0.35	0.09	0.10	0.03	0.01	0.02	0.03	0.02	0.00	0.04
		**Mean ± SEM**	0.73±0.13	0.03±0.02	0.06±0.01	0.02±0.01	0.01±0.00	0.01±0.00	0.02±0.01	0.01±0.00	0.00±0.00	0.02±0.01
**Tomato**	**AB-T**	1	0.00	0.43	0.18	0.09	0.08	0.04	0.02	0.07	0.02	0.00
		2	0.00	0.13	0.10	0.17	0.08	0.04	0.03	0.00	0.30	0.01
		3	0.00	0.11	0.04	0.07	0.03	0.04	0.00	0.00	0.16	0.01
		4	0.00	0.11	0.22	0.03	0.05	0.00	0.01	0.01	0.00	0.01
		**Mean ± SEM**	0.00±0.00	0.20±0.08	0.14±0.04	0.09±0.03	0.06±0.01	0.03±0.01	0.02±0.01	0.02±0.02	0.12±0.07	0.01±0.00
	**AB-UT**	1	0.90	0.01	0.03	0.00	0.00	0.01	0.00	0.00	0.00	0.01
		2	0.63	0.12	0.04	0.03	0.02	0.02	0.00	0.00	0.00	0.00
		3	0.69	0.10	0.04	0.00	0.00	0.04	0.01	0.00	0.00	0.01
		4	0.82	0.01	0.04	0.00	0.00	0.01	0.00	0.00	0.04	0.00
		**Mean ± SEM**	0.76±0.06	0.06±0.03	0.04±0.00	0.01±0.01	0.01±0.01	0.02±0.01	0.00±0.00	0.00±0.00	0.01±0.01	0.01±0.00
